# Key Performance Indicators Related to Strength, Endurance, Flexibility, Anthropometrics, and Swimming Performance for Competitive Aquatic Lifesaving

**DOI:** 10.3390/ijerph18073454

**Published:** 2021-03-26

**Authors:** Daniela Reichmuth, Bjørn Harald Olstad, Dennis-Peter Born

**Affiliations:** 1 Department for Elite Sport, Swiss Federal Institute of Sport Magglingen, 2532 Magglingen, Switzerland; d.reichmuth@slrg.ch; 2 Swiss Lifesaving Swimming Federation, 6210 Sursee, Switzerland; 3 Department of Physical Performance, Norwegian School of Sport Sciences, 0863 Oslo, Norway; bjornho@nih.no; 4 Section for High-Performance Sports, Swiss Swimming Federation, 3063 Berne, Switzerland

**Keywords:** athlete, competition, elite, normative data, swimming, testing

## Abstract

The aim of the study was to investigate key performance indicators for the individual pool-based disciplines of competitive lifesaving regarding strength, flexibility, sprint and endurance swimming performance, anthropometric characteristics, and technical skills specific to competitive lifesaving. Data were collected from Swiss national team members (seven males: age 19 ± 2 yrs, body mass 77 ± 11 kg, body height 177 ± 7 cm and seven females age 21 ± 5 yrs, body mass 64 ± 6 kg, body height 171 ± 4 cm) competing at the 2019 European lifesaving championships. Potential key performance indicators were assessed with race times derived from the 2019 long-course season using Spearman’s correlation coefficient. Large and significant correlations showed that sprint, i.e., 50 m freestyle performance (*r* ≥ 0.770), was related to race time of all pool-based disciplines, rather than endurance swimming performance. Additionally, significant correlations revealed upper body strength, i.e., bench press (*r* ≥ −0.644) and pull (*r* ≥ −0.697), and leg strength (*r* ≥ −0.627) as key performance indicators. Importance of the lifesaving-specific skills, anthropometric characteristics, and core strength varied between the disciplines. Flexibility was not significantly related to race times of competitive lifesaving. The present study showed that sprint swimming performance, upper body, and leg strength are particularly important for competitive lifesaving. As other physical and technical requirements varied between the pool-based disciplines, coaches may use the present key performance indicators to establish training guidelines and conditioning programs as well as prioritize skill acquisition in training to specifically prepare athletes for their main disciplines.

## 1. Introduction

Long coastlines at the ocean, a high number of publicly accessible swimming pools and lakes require many well-trained lifeguards all around the world [1]. Educational programs to become a lifeguard require recruits to swim 500 m in less than 13 min in Switzerland (country of origin of the present study) and 200 m in less than 6 min in Australia (one of the best and most professional lifesaving nation) in addition to various diving and towing tasks [2,3]. However, previous research showed that faster swim times of 3.5 min and 7.5 min for 200 m and 400 m, respectively, improve chances for successful lifesaving actions [4,5]. To motivate lifeguards to continuously improve their physical fitness, competitions with lifesaving-specific disciplines were established [1]. These competitions became a well-recognized sport, especially in the USA, Australia, and New Zealand, with elite athletes professionally training all year around [1]. Although the fitness level of these elite competitive lifesavers may go far beyond the requirements of ordinary lifeguards [4,5], they may serve as role model and motivate lifeguards to establish training routines [6].

Even though lifesaving is a recognized competitive sport, research and data on this sport are sparse. Little is known about contribution of physical and technical abilities required for success in lifesaving competitions. To compete and train all year around, half of the disciplines in international lifesaving competitions are held in long-course (50 m) swimming pools. These pool-based disciplines require competitive lifesavers to swim under immersed obstacles, dive, carry and tow an up to 35 kg heavy manikin, swim with and without fins [7]. Therefore, well-established aerobic and anaerobic endurance may be needed for race distances between 50 m and 200 m [8,9]. In Olympic pool swimmers, the significance of upper-body and core-strength on sprint performance is clear, but controversially discussed for middle- and long-distance performance [10,11]. Competitive lifesaving disciplines involve carrying and towing the manikin either by holding it with one arm, or by attaching it to a lifesaving specific towing device, named rescue tube [7]. Therefore, greater upper body and core strength may be required even in longer race distances to overcome the additional drag forces of the manikin compared to the Olympic swimming disciplines.

Additionally, leg strength and power may be of particular importance for competitive lifesavers. Three pool disciplines in competitive lifesaving involve swimming with fins, which increases mechanical efficiency of swimming by 10% [12]. Here, lifesavers cover the longest distance possible underwater to benefit from the 7% lower drag forces compared to fin swimming at the surface [13]. Holding arms and upper body in a streamline position with as little movement as possible, legs produce the propulsion from hips downward [14]. The approximately 40% lower kicking frequency with fins compared to without fins increases the need for greater lower limb power [12,15].

Flexibility and anthropometric traits may provide further key performance indicators for competitive lifesavers. Specific shoulder, hip, and ankle flexibility are important contributors to performance and injury prevention in Olympic swimmers [16,17,18]. Additionally, successful swimmers are tall, with a large arm span [19] that contribute to low drag and high propelling efficiency [20]. Despite similarities in the physiological profile of competitive lifesavers and Olympic swimmers, technical requirements may differ substantially. For instance, the 100 m manikin carry requires the lifesavers to cross the 50 m pool swimming with fins. Thereafter they pick up the submerged manikin and carry it 50 m back to the other poolside. As most disciplines in competitive lifesaving involve a large variety of skills, such as swimming with and without fins, carrying, and towing a manikin across various distances, a detailed analysis of the sub-disciplines and their contribution to overall lifesaving competition performance is required to establish training guidelines and facilitate long-term athlete development.

As such, the aims of the study were (1) to identify key performance indicators regarding strength, sprint and endurance swimming performance, flexibility, anthropometric characteristics, as well as technical skills specific to competitive lifesaving and (2) to assess their contribution to the individual pool-based disciplines. The hypotheses were that (1) swimming skills would show strong relationships with competitive lifesaving performance and (2) physical and technical skills required would vary between each of the disciplines.

## 2. Materials and Methods

### 2.1. Subjects

Fourteen Swiss national team members (seven males and seven females) competing at the 2019 European lifesaving championships in Riccione, Italy participated in the study. Only athletes with no medical or health-related issues during the study period were included. Six (three females and three males) competed in the junior category. In the championship team ranking, the senior and junior national teams achieved 8th and 10th place among the 20 and 15 participating nations, respectively. The study was pre-approved by the leading institution’s review board (Reg.-Nr. 089LSP19) and is in line with the Code of Ethics of the World Medical Association (Helsinki Declaration). After being informed about the benefits and risks of being involved in the study, all participants or legal guardians for minors provided written informed consent prior to participation.

### 2.2. Study Design

**Figure 1:**
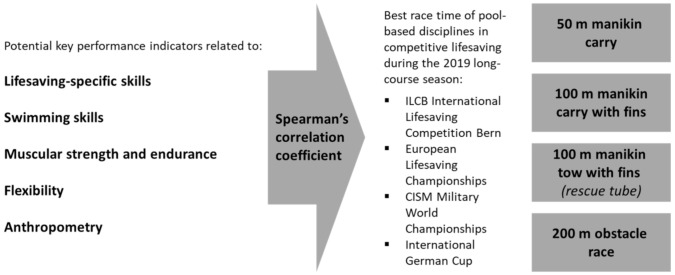
Study design.

Performance data were collected for technical skills of competitive lifesaving, freestyle sprint and endurance swimming, muscular strength and endurance, flexibility, and anthropometric characteristics. In order to investigate contribution to individual pool-based disciplines of competitive lifesaving, i.e., 50 m manikin carry, 100 m manikin carry with fins, 100 m manikin tow with fins (rescue tube), and 200 m obstacle race, best race time for each of the disciplines from the 2019 long-course season (competitions held in 50-m pools) were extracted and correlated with the potential key performance indicators. Only results from official international competitions of the national team members, i.e., ILCB International Lifesaving Competition Bern, European Lifesaving Championships, CISM Military World Championships, and International German Cup, between September and November 2019 were considered after which performance data for determination of key performance indicators were collected (Figure 1).

### 2.3. Data Collection

In order to determine key performance indicators, all in-water- and land-based performance analyses were performed in a standardized order across a nine-hour timespan. Rest periods between the tests were used to assess four athletes interchangeably on the same day and to avoid fatigue. First, strength tests were conducted over a period of three-hours. Then, swimmers underwent various in-water tests on specific competitive lifesaving and usual swimming skills for two hours. After a 90 minute lunch break, 10–15 min of flexibility and anthropometric tests were performed on each athlete. The last test was an incremental endurance swimming test (1.5 h). None of the female athletes were menstruating on the day of testing.

#### 2.3.1. Competitive Lifesaving Skills

All swimming tests were performed in a 50 m long-course pool. Block and 50 m split times were measured with an electronical timing system (Quantum Aquatics, Swiss Timing LTD, Corgémont, Switzerland). The 50 m swimming test with fins began with an official on-block competition start [21] and no limits to the length of the subsequent underwater phase [7]. At the 15 m and 25 m mark, cameras (CX550, Sony Group Corporation, Tokyo, Japan) collected 50 Hz video footages at a right angle to the swimming lane to determine 15 m and 25 m split times. A video analysis software (Kinovea 0.9.1; Joan Charmant & Contrib., kinovea.org), measured the time between the light flash from the starting signal and the top of the head passing the 15 m and 25 m marks. The 25 m manikin carry was performed with an in-water start, which consisted of a push-off from the pool wall. The athletes carried the manikin with one arm and used the other arm for propulsion (single-arm front crawl) [7]. The 50 m manikin tow was performed with a rescue tube and fins, beginning with the in-water start. The rescue tube was attached to the manikin and the swimmer’s upper body before the trial. In line with official rules for the carrying disciplines [7], the manikin was completely filled with water (total weight: 35 kg). For towing tests with the rescue tube, the manikin was filled to the upper line of the white markings and able to float at the surface (total weight: 25 kg).

#### 2.3.2. Swimming Skills

The 50 m swimming test was assessed in the same pool with the same timing system used for the competitive lifesaving skills. To assess freestyle turn performance, athletes swam from 20 m in front of the pool wall, performed a tumble turn and swam back well past the 10 m mark. A video camera positioned at a right angle to the swim lane was used to measure the top of the head passing the 10 m mark before and after wall contact. The best of three attempts, with a 5 min rest period between trials, was used for statistical analysis. Endurance capacity was tested with an incremental step test as described in detail previously [22]. Blood lactate concentration was measured prior to the test and after each 200 m step (LactatePro2, LT-1730, Arkray, Kyoto, Japan). The test involved four swimming velocities aiming for 82, 88, 92, 95% of previous 200 m personal best times, followed by a 200 m all-out attempt to determine maximal swimming velocity and peak lactate concentration. Pacing was controlled by manually measured 50 m split times (Seiko S141, Seiko Watch Corporation, Tokyo, Japan) [23].

#### 2.3.3. Muscular Strength and Endurance

Before assessing muscular strength and endurance, participants performed a standardized warm-up including five minutes of running at moderate intensity followed by 20 air squats and 10–15 push-ups [24,25,26]. Explosive leg strength was determined by a standing long-jump, where participants performed a double-legged jump from a marked starting line with hands on their hips. A valid trial required participants to stand still after landing for at least three seconds with no adjustment of their foot position. Jumping distance was determined from the starting line to the heel of the rearmost foot with an accuracy of one millimeter using a measuring tape (Frame Series Standard, BMI, Hersbruck, Germany). Participants performed two familiarization jumps. Then, the longest out of three jumps was used for statistical analysis. A two-minute rest period between each jump assured full recovery [27].

Specific warm-up before one-repetition maximum (1RM) bench press and pull tests involved 10 repetitions each with 50% of estimated 1RM [24]. Initial 1RMs were estimated from training data, as all athletes perform bench press and pull exercise in their regular strength training. The procedure for both 1RM tests were as follows: the athletes performed a single repetition with 90% of their estimated 1RM five minutes after the warm-up [24]. After successful completion, the weight of each set was increased by 2.5–5 kg until voluntary failure. One unsuccessful attempt per exercise was allowed and the last successful attempt was used for statistical analysis. A three-minute rest period between each set assured full recovery [24]. To assess bench press 1RM, athletes laid supine on a flat bench with feet positioned on a box with the same height as the bench and a 90° knee angle (Hammer Strength Life Fitness Corporate, Rosemont, IL, USA). Arching the back was not allowed and examined by an experienced investigator. Using medium grip width, hand positions were marked with tape during the warm-up to assure optimal force production and standardize testing procedure [24]. A valid attempt required the bar to be lowered down to the chest and pushed back until the elbows were straight and locked. A spotter was positioned behind the athlete for safety reasons but not allowed to intervene for a valid attempt. To assess bench pull 1RM, participants laid prone on a bench with the chin in contact with the top of the bench (Hammer Strength Life Fitness Corporate, Rosemont, IL, USA). Athletes were instructed to grab the bar 2–3 cm wider than shoulder width when arms were extended. During each attempt, legs were held down and secured on the bench by an assistant. A valid attempt required the bar to be pulled from fully extended arms straight up to the bottom of the bench [28].

Core strength was assessed in ventral, lateral, and dorsal body position with a five-minute rest period in-between [25,26]. For all core tests, a metronome determined a one-second rhythm throughout (App Natural Metronome, Single Minded Production LLC, Margate, FL, USA). The athletes received two warnings if contact to one of the reference markers, i.e., crossbar, ground, or wall, was lost. The test was aborted with the third failure to keep the predetermined form or when the rhythm could no longer be maintained. Time for all core strength tests was measured manually with a stopwatch (Seiko S141, Seiko Watch Corporation, Tokyo, Japan). Ventral core strength was assessed in a forearm plank position while lifting the feet alternatively 2–5 cm above the ground to the rhythm of the metronome. Athlete’s back had to keep contact with a crossbar and the top of their head with the wall in front of them throughout the entire attempt. To assess lateral core strength, athletes held a lateral plank position with feet on top of each other and their back and shoulders in contact with the wall behind them. The pelvis was lowered to the ground and lifted back up to a crossbar in rhythm to the metronome. Due to the large test battery, lateral core strength was assessed on the athlete’s preferred side only. To assess dorsal core strength, athletes laid prone with their thighs and hips in contact with a 1.5 m high box. Feet were fixed under a crossbar behind them. The upper body was moved with a straight back between two crossbars from a horizontal position down to 30° hip flexion and back up [25,26].

#### 2.3.4. Flexibility

Flexibility was assessed with one attempt for each test with no stretching allowed beforehand. Athletes were instructed to engage in each position for 10 seconds with their deepest joint angle. To investigate shoulder inward and outward rotation, athletes laid supine with shoulders abducted, elbows flexed at 90°, and fingertips pointed towards the ceiling [29,30]. Forearm rotation in direction of the hips determined inward and in direction of the head outward rotation, respectively. For both movements, elbows were in contact with the ground at shoulder height. Using a goniometer, maximal joint angles were measured (Goniometer KaWe, Kirchner&Wilhelm, Asperg, Germany). Hip extension was determined in a prone position by actively lifting the straight leg as high as possible. A goniometer at the greater trochanter detected changes in the hip angle [29]. For the sit-and-reach test, athletes sat straight legged with the feet at a 90° angle against the side of a 20 cm high box. Hands laid flat on top of the box and were pushed forward. The distance between fingertips and toes determined flexibility [31]. To investigate foot flexibility, athletes sat upright on the floor and extended the foot as far as possible with straight legs and heels in contact with the ground. A goniometer positioned at the knuckle of the ankle determined plantarflexion angle with the fibula head and joint of smallest toe as reference points. Additionally, athletes rotated the foot inward at maximal plantarflexion. Maximal displacement of the joint of the smallest toe from the lateral side of the shank determined supination [29,32].

#### 2.3.5. Anthropometrics

Body height was measured barefoot standing upright with feet, back, and head against the wall with an accuracy of one millimeter with a measuring tape (Frame Series Standard, BMI, Hersbruck, Germany). Arm span was measured with both arms extended sideways with a 90° abduction at the shoulders. Distance between the longest finger of each hand determined arm span. Body mass was assessed barefoot wearing shorts and a t-shirt using a scale (Seca 876, Seca, Hamburg, Germany).

### 2.4. Statistical Analyses

Normality was confirmed with Shapiro–Wilk’s test. Non-normally distributed data showed a Gaussian distribution after logarithmic transformation. As data collection in elite athletes naturally comes with a low sample size when aiming for the highest possible performance level, correlation analyses were performed with a mixed gender approach as suggested previously [33,34]. To account for the low sample size, Spearman’s correlation coefficient was used to correlate best race time for each of the pool-based disciplines from the long-course season with the performance indicators. Coefficients of <0.3, 0.3–0.5, 0.5–0.7, 0.7–0.9, and >0.9 were classified as small, medium, large, very large, and excellent, respectively [35]. An alpha-level of 0.05 indicating statistical significance. Data were prepared with Microsoft Excel 2016 (Microsoft Corporation, Redmond, WA, USA) and analyses carried out using the JASP statistical software package version 0.14 (JASP-Team University of Amsterdam, Amsterdam, The Netherlands) [36].

## 3. Results

Descriptive data of the performance indicators related to strength, sprint and endurance swimming performance, flexibility, anthropometric characteristics, as well as technical skills specific to competitive lifesaving are presented in Table 1. Additionally, data were split by gender to provide comparative data for coaches and athletes in Table A1. Contribution of performance indicators to the pool-based disciplines of competitive lifesaving is presented in Table 2. Lifesaving-specific skills reflected the specific requirements for each of the disciplines, i.e., 50 m manikin carry, 100 m manikin carry with fins, 100 m manikin tow with fins (rescue tube), and 200 m obstacle race. Non-significant correlations revealed that block time was no key performance indicator. Large (*r* ≥ 0.770) and significant correlations (*p* ≤ 0.01) showed that sprint, i.e., 50 m freestyle performance, was related to race time of all four lifesaving disciplines, rather than endurance swimming performance. While significant correlations revealed upper body, i.e., bench press (*r* ≥ −0.644) and pull (*r* ≥ −0.697), and leg strength (*r* ≥ −0.627) as key performance indicators, importance of core strength and anthropometric characteristics varied between the disciplines. Flexibility showed no significant correlation with race time of any of the lifesaving disciplines.

## 4. Discussion

The present study investigated potential key performance indicators regarding strength, flexibility, sprint and endurance swimming performance, anthropometric characteristics, as well as technical skills specific to competitive lifesaving and their contribution to the pool-based disciplines. Correlation analysis revealed sprint swimming performance, upper body and leg strength rather than endurance swimming performance as key performance indicators for the pool-based lifesaving disciplines. However, importance of lifesaving-specific skills, core strength, and anthropometric characteristics depended on the specific requirements of each discipline. Flexibility was not related to race time in any of the disciplines.

### 4.1. Lifesaving-Specific Skills

In the present study, block time was not related to competition performance. This is in line with Olympic pool swimmers, where only small to medium correlations were found between block time and sprint performance [37]. Despite these correlations, the small timely contribution to total race time and the small difference between higher and lower ranked swimmers, block time had no decisive role on race results, thus supporting our findings [37]. The 200 m obstacle race performance showed very large correlations with 50 m freestyle sprint performance. This was surprising, considering the 200 m an endurance rather than sprint event mainly relying on aerobic energy contribution [9]. However, the re-acceleration after diving under each of the eight floating obstacles during the 200 m obstacle race [7] seems to benefit from sprinting abilities.

### 4.2. Swimming Skills

The incremental swimming test is commonly used to assess endurance performance, [22,23]. However, in the present study pace at 4 mmol/L blood lactate concentration showed low correlations with most competition-specific performances, i.e., 50 m manikin carry, 100 m manikin tow with fins, and 200 m obstacle race. Similarly, in elite Olympic swimmers, anaerobic threshold pace determined by blood lactate concentration from an incremental step test also showed weak relationships with competition performance [23], although the incremental step test may still be useful to predict intensity zones for training [22]. However, pool-based disciplines of competitive lifesaving involve a large variety of technical skills. With the high reliance on muscular strength and sprint abilities shown in the present correlation analyses, aerobic endurance capacity is only a single performance contributor among many other important physiological variables. As the incremental step test is fairly time consuming, i.e., 1.5 h, it may be rated with low priority among the other tests when assembling a test battery for competitive lifesavers.

### 4.3. Muscular Strength and Endurance

In Olympic swimmers, the importance of maximal strength on start and sprint performance is clear [11] but controversially discussed for middle and long-distance swimming performance [38]. The present study showed large and significant correlations between upper body and leg strength and competitive lifesaving performance, i.e., 100 m manikin carry and tow with fins. In these disciplines, the necessity of upper body strength may be due to the 25–35 kg manikin that must be moved through the water, creating additional drag forces. Fins involved in these disciplines may further explain associations with leg strength shown in Table 2. While leg kicking mainly contributes to a streamlined body position in freestyle swimming [39], leg muscles generate the major propulsion when swimming with fins [15]. In particular, during 100 m manikin carry and tow with fins, competitive lifesavers first cross the pool before picking up the manikin [7]. This is normally done using only underwater dolphin kicking due to the better movement efficiency under water [13], thus further increasing demand for leg strength [14,40].

### 4.4. Flexibility

From the various competitive lifesaving disciplines, i.e., (fin) swimming in the pool, surf ski and rescue board in the ocean, as well as sprint running on the beach, flexibility may be most important for (fin) swimming [29,32,41]. Therefore, the present test battery was based on common flexibility tests in swimmers [29,30,31,32]. However, limited relationship between flexibility and performance may indicate a ceiling effect of flexibility needed in the pool events of competitive lifesaving. After reaching the required level in flexibility to compete at the European lifesaving championships, further gains may not translate into improved pool swimming performance.

### 4.5. Anthropometrics

Medium to large correlations were shown for taller athletes being faster in the pool-based competitive lifesaving disciplines which is in accordance with previous studies, where body height in particular was related to performance in sprint events [42]. In contrast to previous studies, where lower body mass was associated with lower passive drag forces [43], heavier athletes performed better in the manikin tow in the present study. Due to the resistance caused by the manikin, high propulsive force, hence muscular strength, may be more important than low passive drag forces and optimal streamlining. As muscular strength correlated with performance in the manikin tow, correlations between higher body mass and faster swim times may go back to the higher muscle mass associated with the higher muscular strength needed to transport the 25 kg manikin.

### 4.6. Methodological Considerations

The best individual competition result from each discipline across the long-course season was used to assess contribution of potential performance indicators to the pool-based disciplines. Only the ‘Super lifesaver’ and ‘Rescue medley’ were not included here, as these disciplines combine all of the skills needed in the other pool events, i.e., freestyle swimming, manikin carrying, towing, and fin swimming. In the present test battery, competitive lifesaving specific swimming skills were investigated with fairly short distances, i.e., 25 m and 50 m, to isolate the specific skill set from physiological capacity and endurance performance. Considering the complete 9 hour test battery, it may not be practical to apply all tests in the routine performance analysis procedures of competitive lifesavers. Therefore, performance analysts may choose most relevant performance tests for their athletes based on the present findings (refer to Table 2).

As a population, elite athletes naturally come with low sample size, i.e., members of the national all-star team. To enhance statistical power, athletes from the senior and junior national team were therefore pooled. This was based on the rationale that the six junior athletes had a mean age of 17.0 ± 0.9 yrs and were on the doorstep towards the senior age-category. It is also known from other aquatic sports, i.e., swimming, that athletes perform well at a young age and even outperform older swimmers. For instance, during the 2016 Rio Olympic Games the youngest 100 m freestyle finalist outperformed all other competitors being only 18 years old [44].

## 5. Conclusions

As competitive lifesavers require a large variety of skills, i.e., to swim with and without fins, to carry and tow an up to 35 kg heavy manikin across various distances, assessment of key performance indicators for the pool-based disciplines of competitive lifesaving may facilitate long-term athlete development and help to establish training guidelines and conditioning programs. In particular, freestyle sprint rather than endurance swimming performance are of great importance for competitive lifesaving. Great upper body strength, i.e., bench press and pull, are important to overcome the additional drag forces when moving the manikin through the water. The use of fins in some of the lifesaving disciplines, i.e., manikin carry and tow, increases swimming velocity but places a higher load on leg muscles compared to swimming without fins, hence requiring leg strength. Non-significant correlations indicated a ceiling effect and revealed that flexibility is no key performance indicator. Specific technical lifesaving skills, i.e., swimming with fins, manikin carry and tow, showed high importance for competition performance. However, required skills varied and depended on the specifics of each of the discipline, i.e., 50 m manikin carry, 100 m manikin carry with fins, 100 m manikin tow with fins (rescue tube), and 200 m obstacle race. Based on the present key performance indicators coaches may prioritize skill acquisition in training and prepare athletes for the specific requirements of their main disciplines.

## Figures and Tables

**Table 1:** Descriptive data for the performance indicators.

Performance Indicators	Mean ± Standard Deviation (n = 14)
Lifesaving-specific skills	
Block time fins [s]	0.8 ± 0.1
50 m swimming with fins [s]	23.7 ± 2.4
25 m manikin carry [s]	23.3 ± 3.1
50 m manikin tow with fins [s]	33.7 ± 3.1
Swimming skills	
Block time freestyle [s]	0.7 ± 0.0
50 m freestyle [s]	30.5 ± 2.7
15 m split time [s]	7.8 ± 0.7
25 m split time [s]	14.1 ± 1.2
Tumble turn [s]	12.2 ± 1.0
200 m freestyle [s]	159.7 ± 12.9
Pace at 4 mmol/L [s/100m]	90.6 ± 4.5
Muscular strength and endurance	
Ventral core [s]	307.5 ± 217.7
Lateral core [s]	60.6 ± 20.3
Dorsal core [s]	99.7 ± 17.7
Bench press [kg]	62.6 ± 23.2
Bench pull [kg]	60.0 ± 20.1
Standing long jump [cm]	180.0 ± 19.5
Flexibility	
Shoulder inward rotation [°]	121.8 ± 14.5
Shoulder outward rotation [°]	83.8 ± 12.6
Hip extension [°]	17.0 ± 5.4
Sit-and-reach [cm]	9.0 ± 10.9
Plantar flexion [°]	12.9 ± 6.1
Supination [°]	25.8 ± 8.6
Anthropometry	
Age [yrs]	20.9 ± 4.4
Body height [cm]	175.4 ± 7.5
Arm span [cm]	175.7 ± 8.1
Body mass [kg]	71.4 ± 11.5

**Table 2:** Performance indicators correlated with race times from pool-based disciplines of competitive lifesaving using Spearman’s correlation coefficient (n = 14).

Performance Indicators	50 m Manikin Carry	100 m Manikin Carry with Fins	100 m Manikin Tow with Fins	200 m Obstacle Race
Lifesaving-specific skills				
Block time fins	−0.136	0.000	0.197	0.598
50 m swimming with fins	0.791 **	0.994 ***	0.841 ***	0.929 **
25 m manikin carry	0.909 ***	0.905 **	0.699 *	0.643
50 m manikin tow with fins	0.855 **	0.857 *	0.797 **	0.821 *
Swimming skills				
Block time freestyle [s]	0.200	0.436	0.393	0.000
50 m freestyle [s]	0.770 **	0.905 **	0.848 ***	0.964 **
15 m split time [s]	0.904 ***	0.905 **	0.772 **	0.821 *
25 m split time [s]	0.877 ***	0.898 **	0.875 ***	0.775 *
Tumble turn [s]	0.808 **	0.976 ***	0.857 ***	0.955 ***
200 m freestyle [s]	0.440	0.934 ***	0.465	0.685
Pace at 4 mmol/L [s/100m]	0.330	0.790 *	0.063	0.396
Muscular strength and endurance				
Ventral core [s]	−0.400	−0.762 *	−0.531	−0.571
Lateral core [s]	−0.219	−0.659	−0.510	−0.214
Dorsal core [s]	0.402	0.192	−0.131	0.143
Bench press [kg]	−0.644 *	−0.805 *	−0.885 ***	−0.703
Bench pull [kg]	−0.785 **	−0.732 *	−0.697 *	−0.691
Standing long jump [cm]	−0.627 *	−0.976 ***	−0.776 **	−0.821 *
Flexibility				
Shoulder inside rotation [°]	−0.241	0.214	−0.060	0.000
Shoulder outside rotation [°]	0.000	−0.048	0.357	0.071
Hip extension [°]	−0.009	−0.381	0.244	−0.073
Sit-and-reach [cm]	0.329	0.084	0.091	0.234
Plantar flexion [°]	−0.161	−0.313	−0.335	−0.371
Supination [°]	0.284	0.575	0.293	0.414
Anthropometry				
Age [yrs]	−0.041	0.287	−0.412	−0.216
Body height [cm]	−0.475	−0.455	−0.565	−0.582
Arm span [cm]	−0.542	−0.371	−0.551	−0.571
Body mass [kg]	−0.574	−0.524	−0.767 **	−0.857 *

Notes: * *p* < 0.05, ** *p* < 0.01, ***, *p* < 0.001.

## Data Availability

The data presented in this study are available on request from the corresponding author.

## Appendix A

**Table A1:** Descriptive data (mean ± standard deviation) for the performance indicators.

Performance Indicators	Males (n = 7)	Females (n = 7)
Lifesaving-specific skills		
Block time fins [s]	0.8 ± 0.0	0.8 ± 0.1
50 m swimming with fins [s]	21.9 ± 1.9	25.6 ± 1.1
25 m manikin carry [s]	20.9 ± 1.5	25.8 ± 2.0
50 m manikin tow with fins [s]	31.2 ± 2.2	36.2 ± 1.4
Swimming skills		
Block time freestyle [s]	0.7 ± 0.0	0.7 ± 0.0
50 m freestyle [s]	28.5 ± 0.7	32.5 ± 2.3
15 m split time [s]	7.3 ± 0.2	8.2 ± 0.6
25 m split time [s]	13.2 ± 0.4	14.9 ± 1.1
Tumble turn [s]	11.5 ± 0.5	12.9 ± 1.0
200 m freestyle [s]	153.5 ± 10.8	165.9 ± 12.5
Pace at 4 mmol/L [s/100m]	90.0 ± 4.4	91.3 ± 4.7
Muscular strength and endurance		
Ventral core [s]	389.4 ± 276.2	225.6 ± 103.6
Lateral core [s]	69.4 ± 20.8	51.9 ± 16.7
Dorsal core [s]	95.9 ± 14.4	103.6 ± 20.9
Bench press [kg]	82.1 ± 12.9	43.1 ± 10.5
Bench pull [kg]	77.9 ± 10.7	42.1 ± 3.9
Standing long jump [cm]	194.3 ± 15.8	165.7 ± 9.9
Flexibility		
Shoulder inward rotation [°]	118.3 ± 16.5	125.3 ± 12.3
Shoulder outward rotation [°]	82.0 ± 13.8	85.6 ± 11.9
Hip extension [°]	16.0 ± 6.1	18.0 ± 4.9
Sit-and-reach [cm]	7.4 ± 10.5	10.6 ± 11.8
Plantar flexion [°]	14.1 ± 7.4	11.7 ± 4.8
Supination [°]	22.6 ± 8.6	29.0 ± 8.0
Anthropometry		
Age [yrs]	20.7 ± 4.2	21.1 ± 4.9
Body height [cm]	179.3 ± 8.2	171.4 ± 4.4
Arm span [cm]	180.1 ± 8.2	171.3 ± 5.4
Body mass [kg]	78.8 ± 11.0	64.1 ± 6.3
